# Life’s Last Sight

**Published:** 1857-02

**Authors:** 


### LIFE'S LAST SIGHT.

Recent scientific observation is cumulative of the fact, that the last thing seen by those who are instantaneously killed, is painted with perfect minuteness and correctness on the back part of the eye. And, perhaps, if the *retina* is examined immediately after death, in case of murder, the image of the murderer may be distinctly seen.

Dr. Thomas Jefferson Hall, a gentleman of singular personal piety and professional skill, at the moment of dying, raised his hands and called upon his mother and minister, both of whom were dead. It is no unusual thing for the departing to mention some loved name gone before, while angels seem to hover around the good, as if ready to bear them upward towards their heavenly home. Who knows but, if some skilful dissector could be present and could make the examination at the next instant succeeding dissolution, that delicate canvas, prepared and painted by divinity, might not foreshadow "*things unutterable!*" might not give the long and earnestly coveted glimpse—coveted by multitudes—of something *beyond* that "bourne whence no traveller returns!!"

